# Plasma neuropilin-1 is associated with a favorable lipid profile and insulin sensitivity in a population-based sample of 50-year-old individuals

**DOI:** 10.1038/s41598-026-65317-7

**Published:** 2026-08-05

**Authors:** Emanuel Fryk, Vagner Ramon Rodrigues Silva, Marco Bauzá-Thorbrügge, Lena Strindberg, Joel Kullberg, Håkan Ahlström, Martin Wabitsch, Pamela Fischer-Posovszky, Rosie Perkins, Milica Vujicic, Ingrid Wernstedt Asterholm, Lars Lind, Per-Anders Jansson

**Affiliations:** 1 Department of Molecular and Clinical Medicine, Institute of Medicine, Sahlgrenska Academy, University of Gothenburg, Gothenburg, Sweden; 2 Department of Surgical Sciences, Uppsala University, Uppsala, Sweden; 3 Department of Surgical Sciences, SciLifeLab, Uppsala University, Uppsala, Sweden; 4 Antaros Medical AB, BioVenture Hub, Mölndal, Sweden; 5 Department of Pediatric and Adolescent Medicine, Division of Pediatric Endocrinology and Diabetes, Ulm University Medical Center, Ulm, Germany; 6 German Center for Child and Adolescent Health (DZKJ), Partner Site, Ulm, Germany; 7 Department of Physiology/Metabolic Physiology, Institute of Neuroscience and Physiology, Sahlgrenska Academy, University of Gothenburg, Gothenburg, Sweden; 8 Department of Medical Sciences, Uppsala University, Uppsala, Sweden; 9 Region Västra Götaland, Gothia Forum, Sahlgrenska University Hospital, Gothenburg, Sweden

**Keywords:** Soluble neuropilin-1, Adipocytes, Lipolysis, Insulin sensitivity, Epidemiology, Biochemistry, Biomarkers, Diseases, Endocrinology, Physiology

## Abstract

Membrane-bound neuropilin-1 (Nrp-1) has been implicated as a regulator of insulin resistance-related lipid metabolism, but little is known about its circulating form, soluble Nrp-1 (sNrp-1), in a metabolic context. Here we investigated the association between plasma levels of sNrp-1 and metabolic variables in 501 participants from the Prospective investigation of Obesity, Energy and Metabolism (POEM) study. Metabolic phenotyping included blood sampling, dual energy X-ray absorptiometry scan, magnetic resonance imaging for fat distribution, and a glucose tolerance test. Associations were examined using linear regression models and Spearman’s rank correlation. Plasma sNrp-1 levels were inversely associated with levels of total fatty acids, triglycerides and lactate, and positively associated with markers of insulin sensitivity. In human Simpson-Golabi-Behmel Syndrome (SGBS) adipocytes, we showed that treatment with recombinant Nrp-1 reduced phosphorylation of hormone-sensitive lipase in the basal state and attenuated isoproterenol-stimulated glycerol release, a measure of total lipolytic activity. Based on these findings, we thus postulate that sNrp-1-mediated suppression of lipolysis could explain our observed association between plasma sNrp-1 and a favorable lipid profile in humans. Further studies are warranted to establish causality and to clarify the role of sNrp-1 in promoting insulin sensitivity in human adipose tissue.

**Supplementary Information:**

The online version contains supplementary material available at 10.1038/s41598-026-65317-7.

## Introduction

Neuropilin-1 (Nrp-1) is a pleiotropic cell surface receptor^1^ that is highly expressed in endothelium, immune cells and mesenchymal stem cells^1,2^. Increasing evidence supports a role for Nrp-1 in a metabolic context. In endothelial cells, Nrp-1 functions as a co-receptor for vascular endothelial growth factor B (VEGF-B) together with VEGF receptor 1 to promote lipid uptake, transport and storage^2,3^. Adipose tissue Nrp-1 mRNA levels have been shown to correlate positively with the ability of insulin to suppress lipolysis in adipose tissue and insulin sensitivity in humans^2^ and to play a pivotal role in maintaining a healthy weight and glucose tolerance in mice^4^.

Nrp-1 exists not only as a full-length membrane-bound isoform but also as a circulating soluble isoform generated by proteolytic cleavage and alternative splicing of the Nrp-1 gene^5,6^. Evidence in vitro and from animal studies indicates that sNrp-1 acts as a decoy receptor for VEGF receptor ligands, primarily sequestering VEGF and thereby modulating VEGF signaling in endothelial cells^5,7,8^. To date, most studies on sNrp-1 have been performed in the cancer field and whether it also plays a role in metabolism remains unclear.

Here we explored the relationship between plasma sNrp-1 levels and metabolic markers in a population-based setting and the effect of recombinant Nrp-1 (rNrp-1) on lipolysis in human adipocytes.

## Methods

### Study participants

This study evaluated circulating Nrp-1 levels in 501 participants, all aged 50 years, from the population-based Prospective investigation of Obesity, Energy and Metabolism (POEM) cohort in Uppsala, Sweden. The clinical characteristics and further details of this cohort have been described earlier^9,10^. Briefly, participants were examined for anthropometric measures and underwent a 2-h 75 g oral glucose tolerance test (OGTT).

### Body composition analyses

A dual-energy X-ray absorptiometry scanner (Lunar Prodigy, GE Healthcare, USA) was used to measure total fat mass and lean body mass^11^. Whole-body quantitative water-fat magnetic resonance imaging was performed using a 1.5 T clinical MR system (Philips Achieva, Philips Healthcare, Best, Netherlands). Whole-body voxel-wise analysis was used to quantify visceral adipose tissue (VAT) and subcutaneous adipose tissue (SAT) and fat content in the liver and pancreas^12,13^.

### sNrp-1 and other biochemical analyses

Plasma samples were obtained from participants after an overnight fast and analyses were performed according to standard laboratory protocols (Department of Clinical Chemistry, Uppsala University Hospital, Sweden). C-peptide levels were measured using ELISA (Mercodia, Uppsala, Sweden; coefficient of variation (CV) < 5%). sNrp-1 levels were also measured using ELISA (R&D Systems, USA; intra-assay and inter-assay CV 5.3% and 6.0%, respectively).

### Nightingale metabolomics analysis

Metabolomics was performed on fasting plasma samples using the Nightingale metabolomics platform (Nightingale Health, Helsinki, Finland)^14^. Our study concentrated on estimates of lipoprotein fractions, fatty acids and lactate.

### Simpson-Golabi-Behmel Syndrome (SGBS) adipocyte culture

SGBS cells were provided by Prof. Wabitsch, Ulm, Germany. These cells are subcutaneous preadipocytes that have been shown to maintain their capacity for adipogenic differentiation over extended cell cycles. They are a commonly used model for studies of human adipocyte metabolism, and have been extensively characterized and reviewed previously^15^.

SGBS cells were cultured in 12-well plates and differentiated following a standardized protocol^16^. In brief, SGBS preadipocytes were cultured to near confluence in Dulbecco’s Modified Eagle Medium/Nutrient Mixture F-12 (DMEM/F-12; Invitrogen, #31,330–038) supplemented with biotin (3.3 µM, Sigma-Aldrich, #B-4639), pantothenate (1.7 µM, Sigma-Aldrich, #P-5155), penicillin/streptomycin (100 U/mL, Thermo Fisher Scientific, #15,140–122), and fetal bovine serum (Thermo Fisher Scientific, #10,270–106). The medium was then replaced with a differentiation cocktail containing cortisol (0.1 µM, Sigma-Aldrich, #H-0888), apo-transferrin (0.01 mg/mL, Sigma-Aldrich, #T-2252), triiodothyronine (0.2 nM, Sigma-Aldrich, #T-6397), insulin (20 nM, Thermo Fisher Scientific, #12,585–014), rosiglitazone (2 µM, Cayman Chemical, #71,740), dexamethasone (25 nM, Sigma-Aldrich, #D-1756), and 3-isobutyl-1-methylxanthine (IBMX, 250 µM, Sigma-Aldrich, #I-5879) for 4 days to initiate differentiation. Thereafter, differentiation was maintained without addition of rosiglitazone, dexamethasone or IBMX. The medium was changed every four days and experiments were performed on differentiation-day 14.

### Immunoblotting and lipolysis experiments

The SGBS cells were washed twice with PBS and starved in DMEM with glucose 5.6 mM (HyClone, #SH30021) to mimic physiological fasting conditions. Media was supplemented with penicillin/streptomycin (1%) and supplemented with pantothenate (1.7 mM) and biotin (3.3 mM) 24 h before the addition of human rNrp-1 (Catalog #: 3870-N1, R&D Systems, USA).

For immunoblotting, the cells were treated with rNrp-1 (20–2000 ng/mL) added in DMEM with glucose 5.6 mM containing 2% BSA for 30 min. Immediately afterwards, the cells were harvested for protein lysate extraction using RIPA Lysis and Extraction Buffer (Thermo Scientific, Cat#89,900) with Halt™ Protease and Phosphatase Inhibitor Cocktail, EDTA-free (100X) (Thermo Scientific, Cat#78,443). Western blot analysis was performed as described previously^17^. Primary and secondary antibodies were from Cell Signaling (MA, USA): phospho-HSL (Ser660) antibody, #45,804 (1:1.000); HSL antibody, #4107 (1:1.000); anti-rabbit IgG, HRP-linked antibody, #7074 (1:1.000); and phospho-Akt (Ser473), #9271 (1:1.000). The total Akt antibody was from Thermo Fisher Scientific (Invitrogen AKT Pan Monoclonal Antibody, # MA5-14,916 (1:1.000).

Glycerol release experiments were done separately. The cells were treated for 4 h with vehicle or rNrp-1 (2000 ng/ml) and/or isoproterenol (25 nmol/L) added in DMEM supplemented with glucose 5.6 mM and 2% fatty acid-free BSA. After treatment, the plates were immediately placed on ice to stop the reaction. Glycerol concentrations in the media were analyzed according to the Glycerol-Glo™ Assay Technical Manual, TM 599 (Promega J3150 and J3151) as previously reported^18^.

### Statistics

Linear regression was performed using Statistical Package for the Social Sciences (SPSS), IBM SPSS Statistics, Armonk, New York, USA. In the primary analyses, all linear models were adjusted for sex, education, smoking and exercise habits. In secondary sensitivity analyses, an additional adjustment for BMI was added. Non-normally distributed variables were transformed using the natural logarithm. All variables were z-transformed to allow comparison of effect sizes across variables. Spearman’s rank correlation coefficients were subsequently calculated in a sensitivity analysis. To reduce the burden of multiple testing and preserve statistical power in this unadjusted analysis, only total triglycerides and total fatty acids were examined as markers of lipid metabolism. Two-way ANOVA with Dunnett´s or Tukey’s post hoc test were applied for comparisons of experimental results by using GraphPad Prism version 10, San Diego, California, USA. P < 0.05 was considered statistically significant.

## Results

### sNrp-1 measurements

**Fig. 1:**
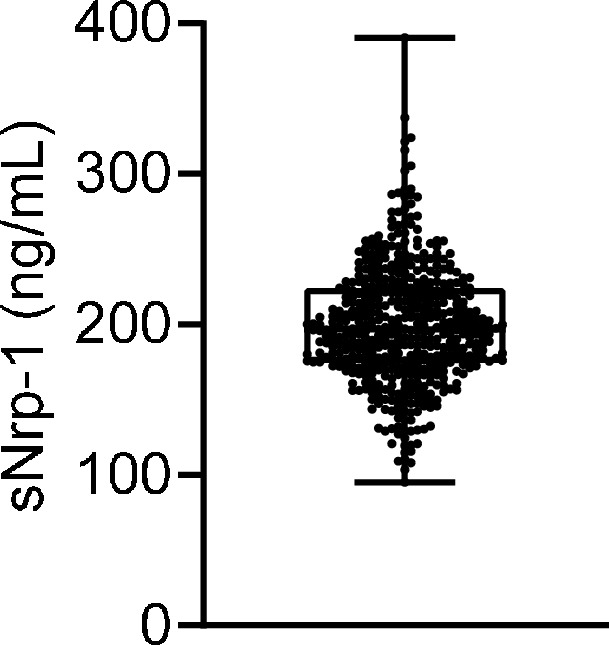
Box and whisker plot showing the distribution of absolute sNrp-1 concentrations in the POEM cohort. Whiskers indicate the maximum and minimum values.

sNrp-1 concentrations ranged between 95 and 390 ng/mL, with a median of 197 ng/mL. The 25^th^ percentile was 174 ng/mL and the 75^th^ percentile 224 ng/mL. Distribution was near normal, but skewed slightly to higher values (Fig. 1).

### Association between plasma sNrp-1 and body composition variables

Plasma sNrp-1 levels inversely correlated with VAT mass but were not associated with any other markers of body composition (Table 1). Inverse associations of plasma sNrp-1 with fat mass and liver fat in BMI-adjusted analyses were borderline significant (*p* = 0.054 and *p* = 0.057, respectively). The significant association observed for VAT mass in the linear regression analysis was not confirmed in the sensitivity analysis using Spearman’s rank correlation (Supplementary Table 1).

**Table 1:** Linear association for plasma soluble neuropilin-1 with body composition and metabolic variables.

	β (CI)	*p* value	BMI-adjusted β (CI)	adj.* p* value
Body composition				
Weight	0.001 (−0.001 : 0.003)	0.373	0.000 (−0.001 : 0.001)	0.393
Body mass index	0.001 (−0.002 : 0.003)	0.596	-	-
Fat mass	−0.001 (−0.002 : 0.001)	0.530	−0.001 (−0.002 : 0.000)	0.054
Waist-to-hip ratio	−0.001 (−0.003 : 0.001)	0.270	−0.001 (−0.003 : 0.000)	0.140
VAT*^†^	−0.003 (−0.005 : −0.001)	0.021	−0.003 (−0.005 : −0.002)	< 0.001
SAT*^†^	0.000 (−0.003 : 0.002)	0.741	−0.001 (−0.003 : 0.001)	0.195
Liver fat*^‡^	−0.002 (−0.005 : 0.001)	0.172	−0.002 (−0.005 : 0.000)	0.057
Pancreas fat*^‡^	0.000 (−0.003 : 0.003)	0.995	0.000 (−0.003 : 0.003)	0.904
Fat-free mass	0.001 (−0.001 : 0.002)	0.268	0.001 (−0.001 : 0.002)	0.413
Glucose metabolism				
Fasting blood glucose	0.001 (−0.002 : 0.003)	0.526	0.001 (−0.002 : 0.003)	0.560
Blood glucose_120_	−0.001 (−0.003 : 0.002)	0.542	−0.001 (−0.003 : 0.002)	0.523
Lactate^#^	−0.0029 (−0.0051; −0.0007)	< 0.001	−0.003 (−0.0052; −0.0008)	0.007
C-peptide	−0.002 (−0.004 : 0)	0.060	−0.002 (−0.004 : 0.000)	0.016
Fasting serum insulin*	−0.003 (−0.006 : −0.001)	0.004	−0.003 (−0.006 : −0.001)	0.003
HOMA*	−0.004 (−0.006 : −0.002)	**0.001**	−0.004 (−0.006 : −0.002)	** < 0.001**
Matsuda index*	0.004 (0.002 : 0.006)	**0.001**	0.004 (0.002 : 0.006)	** < 0.001**
Lipid metabolism				
Cholesterol	0.001 (−0.002 : 0.003)	0.683	0.000 (−0.002 : 0.003)	0.705
HDL-cholesterol	0.004 (0.002 : 0.006)	**0.001**	0.004 (0.002 : 0.006)	** < 0.001**
LDL-cholesterol	0.001 (−0.002 : 0.003)	0.606	0.001 (−0.002 : 0.003)	0.638
VLDL-cholesterol^#^	−0.003 (−0,005: −0,001)	**0.003**	−0.003 (−0,005: −0,001)	**0.002**
Total triglycerides*	−0.005 (−0.007 : −0.003)	** < 0.001**	−0.005 (−0.007 : −0.003)	** < 0.001**
HDL-triglycerides^#^	−0.004 (−0.007: −0.002)	** < 0.001**	−0.005 (−0.007: −0.002)	** < 0.001**
LDL-triglycerides^#^	−0.002 (−0.005: 0.000)	**0.034**	−0.003 (−0.005: 0.000)	**0.022**
VLDL-triglycerides^#^	−0.005 (−0.007: −0.003)	** < 0.001**	−0.006 (−0.007: −0.004)	** < 0.001**
Total fatty acids^#^	−0.002 (−0.004: 0.000)	**0.044**	−0.002 (−0.005: 0.000)	**0.029**
Saturated fatty acids^#^	−0.002 (−0.005: 0.000)	**0.039**	−0.003 (−0.005: 0.000)	**0.024**
Mono-unsaturated fatty acids^#^	−0.003 (−0.006: −0.001)	**0.002**	−0.004 (−0.006: −0.002)	**0.001**
Poly-unsaturated fatty acids^#^	−0.001 (−0.003: 0.002)	0.538	−0.001 (−0.003: 0.001)	0.493

Linear regression adjusted for sex, education, smoking, and exercise habits (left) and additional adjustment for BMI (right). All analyses were done on z-transformed variables to allow for comparison of effect sizes. Statistical significance was defined as *p* < 0.05 (indicated in bold text). *Data were log-transformed using the natural logarithm. ^†^n = 324, ^‡^ n = 309. ^#^Analyzed with Nightingale metabolomics. BMI – body mass index, HDL—high-density lipoprotein, HOMA—homeostatic model assessment, LDL—low-density lipoprotein, SAT—subcutaneous adipose tissue, VAT—visceral adipose tissue, VLDL—very-low density lipoprotein.

### Association between plasma sNrp-1 and glucose metabolism variables

Elevated plasma sNrp-1 was also linked to lower fasting serum insulin levels, reduced HOMA scores, and an increased Matsuda index (Table 1). Further, there was an inverse association between plasma sNrp-1 and C-peptide after adjustment for BMI. Plasma sNrp-1 associated with lower lactate levels whilst there was no association with fasting plasma glucose or glucose levels 2 h after the OGTT (Table 1). The significant associations between plasma sNrp-1 and fasting serum insulin, HOMA score, Matsuda index and lactate were supported by significant Spearman’s rank correlations (Supplementary Table 1) and are illustrated in Supplementary Fig. 1. In contrast, the association with C-peptide was not confirmed, although it approached statistical significance (*p* = 0.053).

### Association between plasma sNrp-1 and lipid metabolism variables

Plasma sNrp-1 was associated with high-density lipoprotein cholesterol and inversely associated with very low-density lipoprotein cholesterol but did not associate with total or low-density lipoprotein cholesterol (Table 1). Additionally, plasma sNrp-1 was inversely associated with triglycerides (total and in each lipoprotein fraction), fatty acids (FA) (total, saturated and monounsaturated but not polyunsaturated) (Table 1). The significant associations between plasma sNrp-1 and total triglycerides and total FA were supported by significant Spearman’s rank correlations (Supplementary Table 1) and are illustrated in Supplementary Fig. 1. The association with high-density lipoprotein cholesterol was not confirmed (Supplementary Table 1).

### rNrp-1 decreases lipolysis in SGBS human adipocytes

**Fig. 2:**
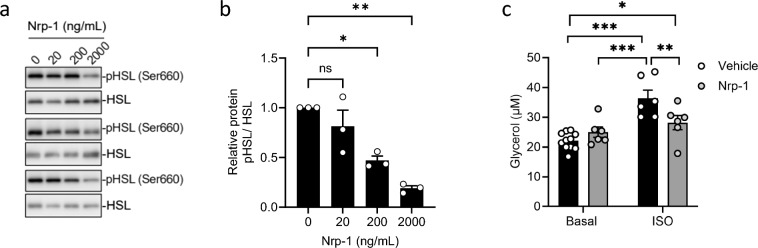
Nrp-1 decreased HSL phosphorylation and isoproterenol-stimulated glycerol release in SGBS-derived human mature adipocytes. (**a**) Western blot and (**b**) corresponding quantification of HSL phosphorylation at Ser660 after stimulation with recombinant Nrp-1 at the indicated concentration for 30 min (n = 3 per group; uncropped blots shown in Supplementary Fig. 2). (**c**) Glycerol release after stimulation with recombinant Nrp-1 (2000 ng/mL) with or without isoproterenol (ISO; 25 nmol/L) for 4 h in SGBS-derived human mature adipocytes (n = 6–11 per group). Nrp-1 was added 5 min prior to ISO. Data are represented as mean ± SEM. Statistical significance indicated as *p < 0.05; **p < 0.01; ***p < 0.001; ns = non-significant; using repeated measures ANOVA with Dunnett´s post-hoc test (**b**) or two-way ANOVA with Tukey´s post-hoc test (**c**).

We observed a dose–response relationship between rNrp-1 (20–2000 ng/mL) and phosphorylation of hormone-sensitive lipase (HSL) at Ser660 in SGBS human adipocytes in the basal state (Fig. 2a and b and Supplementary Fig. 2), whereas no effect was observed on AKT phosphorylation at Ser473 (Supplementary Figs. 2 and 3). Treatment with 2000 ng/mL rNrp-1 suppressed isoproterenol-stimulated glycerol release (Fig. 2c).

## Discussion

In a population-based cohort study of 50-year-old individuals, we observed that higher plasma sNrp-1 levels were linked to a favorable metabolic phenotype with lower levels of plasma triglycerides and FA and increased insulin sensitivity. In support of a functional role of sNrp-1, we showed that rNrp-1 suppressed lipolysis in SGBS-derived human adipocytes.

The adipose tissue plays an important role in regulating the balance of circulating lipids and overall energy balance, to protect against ectopic fat accumulation and insulin resistance^19^. In our human cohort, high plasma sNrp-1 levels were linked to improved insulin sensitivity (lower insulin levels, lower HOMA and higher Matsuda index). However, plasma sNrp-1 was not associated with body weight, total adipose tissue mass, or visceral adipose tissue mass. These findings suggest that the relationship between sNrp-1 and insulin sensitivity is not explained by differences in overall or visceral adiposity and may instead reflect other mechanisms influencing metabolic health.

Lower circulating FA levels are known to be paralleled by lower insulin resistance and insulin secretion independent of changes in glucose levels^20^. Consistent with this, we observed an inverse relationship between plasma levels of sNrp-1 and circulating FA but no association between sNrp-1 levels and fasting glucose or glucose 2 h after an OGTT. Thus, the relationship between sNRP-1 and insulin resistance markers is more closely linked to lipid metabolism than to glucose homeostasis. We also observed an inverse association between sNrp-1 and lactate in plasma. It is possible that this association could be explained by changes in fuel utilization in energy-producing cells^21^.

We hypothesize that our observation of an inverse relationship between sNrp-1 levels and circulating FA, lactate and insulin resistance in the POEM cohort may reflect a role for sNrp-1 in suppressing lipolysis. In the fasting state, HSL plays a key role in maintaining elevated free FA levels through lipolysis while suppressing lipid uptake in adipocytes^19^. In support of our hypothesis, we showed in vitro that rNrp-1 suppressed lipolysis through HSL in the basal state. We also showed that rNrp-1 reduced isoproterenol-stimulated glycerol release, a measure of overall lipolytic activity. In contrast, rNrp-1 did not alter AKT phosphorylation in the basal state, suggesting that its anti-lipolytic effects may be mediated independently of canonical insulin signaling. In a mouse model of diabetes, an earlier study reported that suppression of VEGF signaling in white adipose tissue, and decreased activation of HSL and lipolysis, conferred protection from non-alcoholic fatty liver disease, lipid toxicity and insulin resistance^22^. Given that sNrp1 has been shown to act as an antagonist of VEGF_165_ in tumor cells^5^, we postulate that sNrp-1 could also reduce activation of lipolysis in human adipose tissue by acting as a decoy receptor for VEGF. However, although several associations between sNrp-1 and metabolic variables were statistically significant in our human cohort, their effect sizes were modest. Plasma sNrp-1 is therefore unlikely to be a major determinant of metabolic variation on its own and should be interpreted within the context of the many factors that contribute to metabolic disease.

A limitation of the present study is that the analyses were not adjusted for potential metabolic confounders. Given the modest sample size, including multiple correlated metabolic variables in the linear regression analyses would have increased the risk of overfitting. In addition, the studied variables are biologically interconnected and often exhibit bidirectional relationships, making it difficult to distinguish confounders from mediators or colliders. Consequently, adjustment may have introduced overadjustment bias rather than reducing confounding. Larger longitudinal studies are needed to clarify the causal relationships and identify appropriate adjustment strategies. It should also be noted that the number of cell-based experiments, including appropriate controls, was limited. Further mechanistic studies incorporating additional controls and complementary experimental approaches are needed to validate and extend our findings from these studies.

In conclusion, we show that plasma sNrp-1 associates with a beneficial circulating lipid profile and increased insulin sensitivity in humans and that rNrp-1 suppresses HSL phosphorylation in human adipocytes. Our observations warrant further studies to assess the role of sNrp-1 in promoting insulin sensitivity in human adipose tissue.

## Supplementary Information

Supplementary Information.

## Data Availability

Some raw data analyzed in the present study are not publicly available due to restrictions in handling sensitive personal data, but the data can be accessed through the corresponding author upon reasonable request and with permission of the POEM study steering committee.
