# Supplementary material for: Plasma neuropilin-1 is associated with a favorable lipid profile and insulin sensitivity in a population-based sample of 50-year-old individuals
Source: Sci Rep. 2026 Aug 5;16:24169. doi: 10.1038/s41598-026-65317-7 (PMC13442846; doi:10.1038/s41598-026-65317-7)
Supplement: Supplementary file 1 [file 41598_2026_65317_MOESM1_ESM.pdf]

Supplementary Material

Supplementary Table 1 Spearman's rank correlations for plasma soluble neuropilin-1 with body composition and metabolic variables

	r_s	*p*-value
**Body composition**		
Waist-to-hip ratio	-0.03	0.483
VAT†	-0.03	0.581
SAT†	-0.09	0.111
Liver fat‡	-0.06	0.259
**Glucose metabolism**		
Fasting blood glucose	-0.02	0.628
Lactate#	-0.11	**0.018**
C-peptide	-0.09	0.053
Fasting serum insulin	-0.15	**<0.001**
HOMA	-0.15	**<0.001**
Matsuda index	0.17	**<0.001**
**Lipid metabolism**		
Cholesterol	0.01	0.872
HDL-cholesterol	0.01	0.104
LDL-cholesterol	0.04	0.410
Total triglycerides	-0.18	**<0.001**
Total fatty acids#	-0.10	**0.020**

Statistical significance was defined as $p < 0.05$ (indicated in bold text)

†n=324, ‡ n=309

#Analyzed with Nightingale metabolomics.

Abbreviations: HDL - high-density lipoprotein, HOMA - homeostatic model assessment, LDL - low-density lipoprotein, SAT - subcutaneous adipose tissue, VAT - visceral adipose tissue.

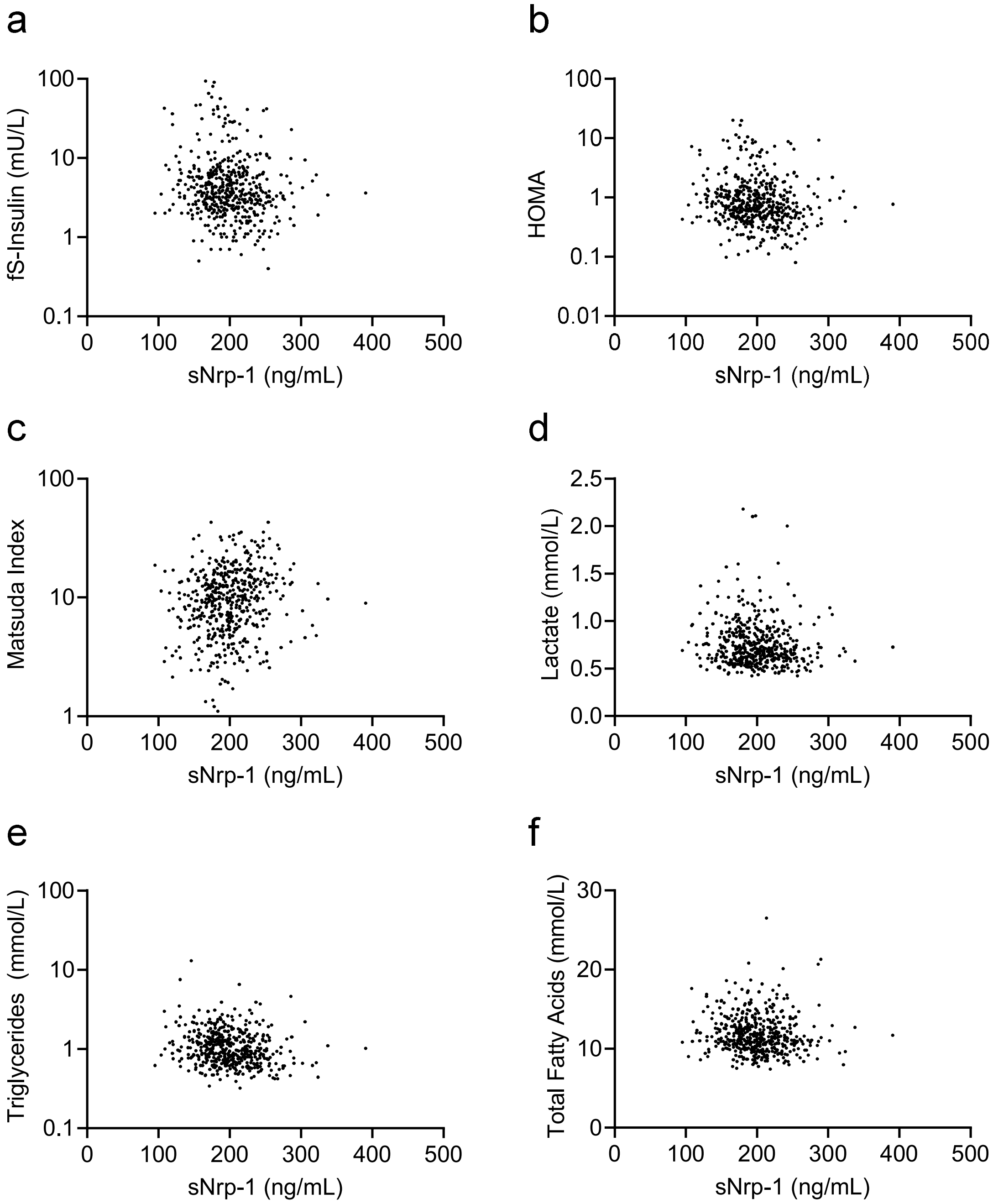

Supplementary Figure 1. Scatter plots for all variables that were significantly associated with sNrp-1 in the linear regression analyses and remained significant in the unadjusted non-parametric Spearman's rank correlation. The y-axes for fasting serum (fS)-insulin, HOMA and Matsuda index use logarithmic scales to improve data visualization.

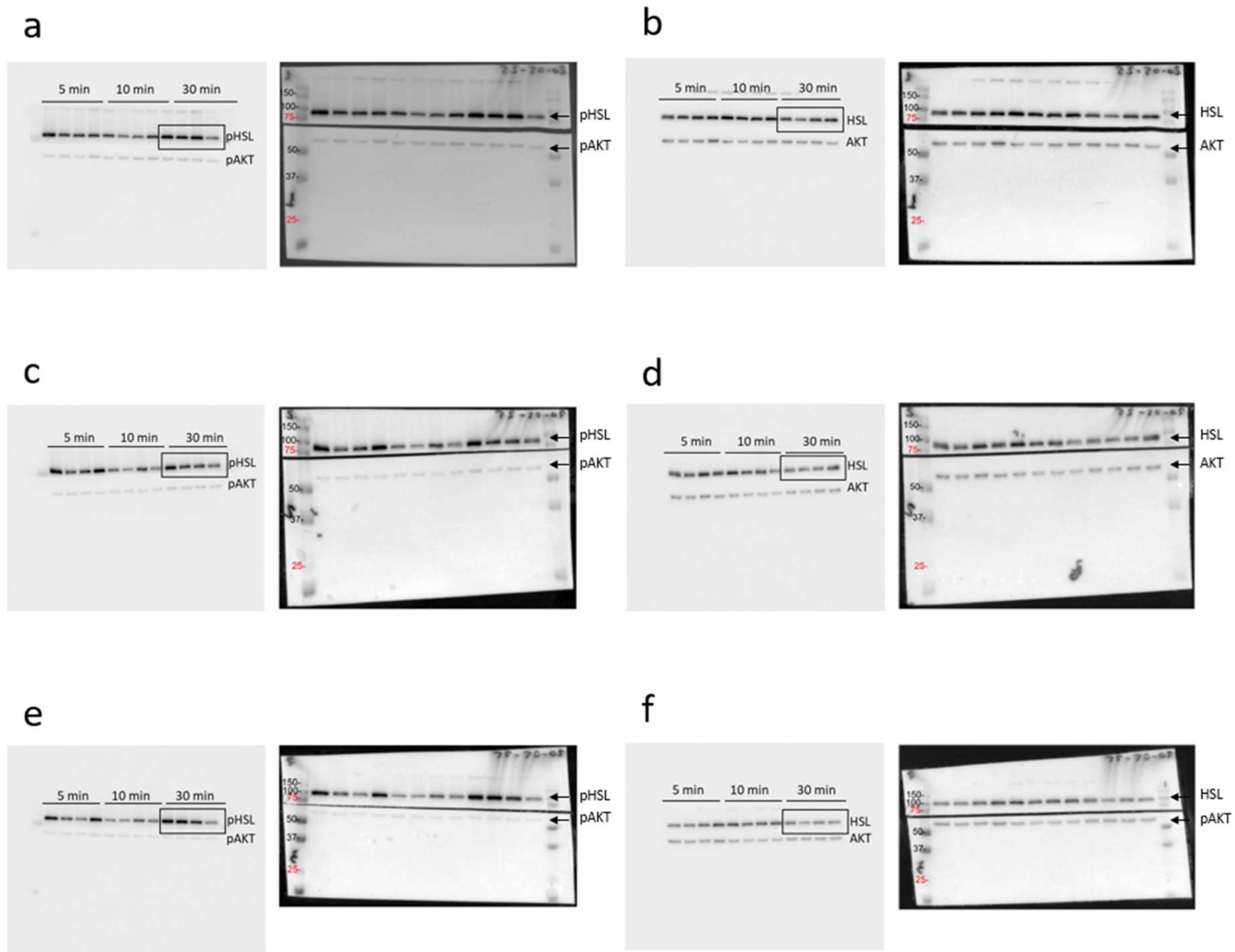

Supplementary Figure 2. Uncropped images of western blot used for quantification (left), as well as blots merged with photo of corresponding membrane with molecular markers for each panel (right) presented in Figure 2a. Figures have consistently been flipped horizontally to compensate for inversion of gel during transfer procedure. Western blots showing HSL phosphorylation at Ser660 after treatment with recombinant Nrp-1 at concentrations 0, 20, 200 and 2000 ng/mL from left to right for each time indicated (5, 10 and 30 minutes). Lower portion of the gel was used to blot AKT phosphorylation at Ser 473 (no significant difference on treatment; Supplementary Figure 3). The membrane was stripped and reprobed to detect total HSL and AKT. The uncropped blots are presented in the same order as in Figure 2a (i.e., blots a and b = rows 1 and 2; blots c and d = rows 3 and 4; and blots e and f = rows 5 and 6). The rectangle in each blot indicates the section presented in Figure 2a.

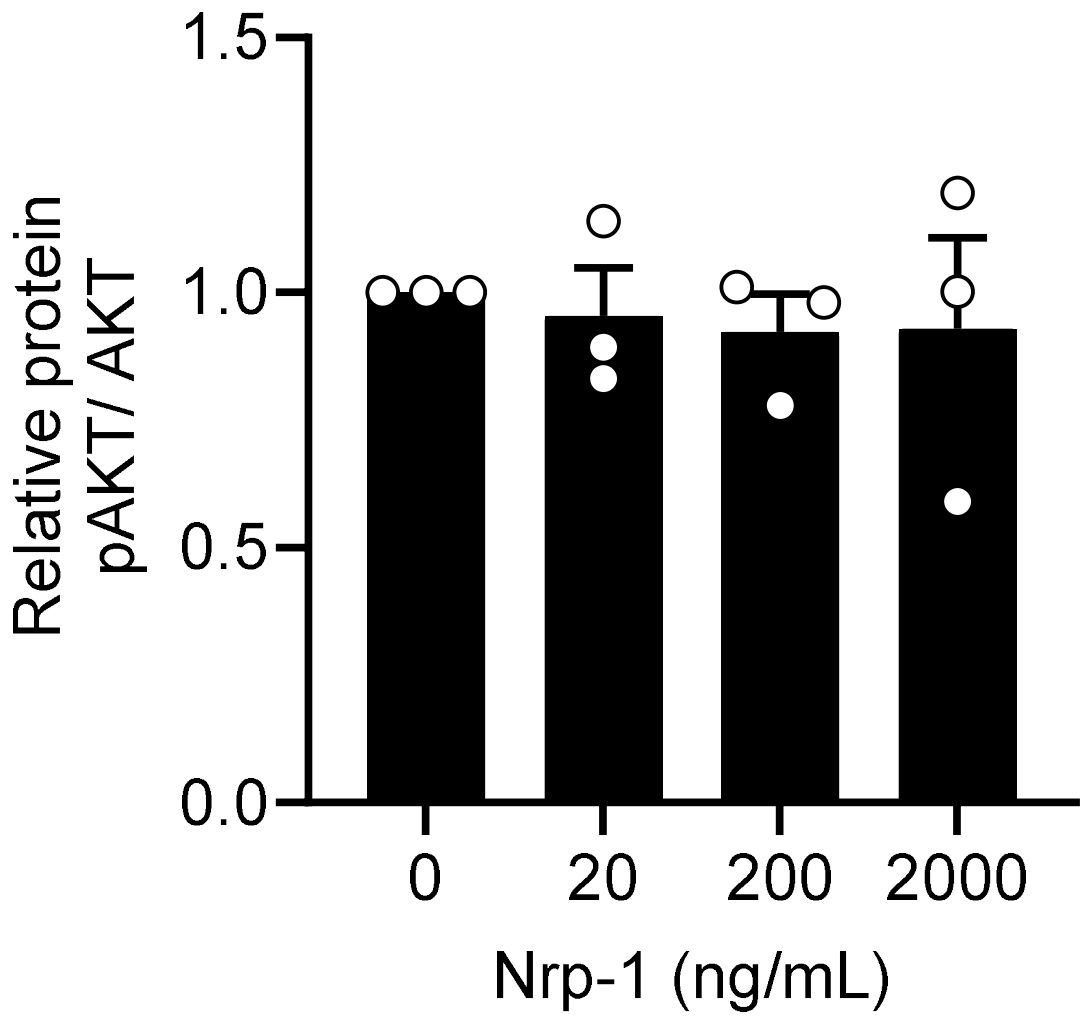

Supplementary Figure 3. Nrp-1 did not affect AKT phosphorylation in SGBS-derived human mature adipocytes. Quantification of western blot of AKT at Ser473 after stimulation with recombinant Nrp-1 at the indicated concentration for 30 minutes (n=3 per group; uncropped blots shown in Supplementary Figure 2). Statistics determined with repeated measures ANOVA with Dunnett´s post-hoc test.
